# Experimental Determination of Wax Appearance Temperature (WAT) in Brazilian Presalt Petroleum via HPμDSC: Effects of Pressure and Gas Composition

**DOI:** 10.1021/acsomega.5c06286

**Published:** 2026-01-20

**Authors:** Marcelo Tai, Roberto Carlos G. de Oliveira, Érika A. Carvalho, Thais L. de Carvalho, Electo E. Silva Lora

**Affiliations:** † Instituto de Engenharia Mecânica (IEM), Universidade Federal de Itajubá (UNIFEI), Av. B P S, 1303–Pinheirinho, Itajubá, Minas Gerais 37500-903, Brazil; ‡ Centro Tecnológico para o Pré-sal Brasileiro (CTPB), Universidade Federal de Itajubá (UNIFEI), Av. Dr. Jerson Dias - Itajuba, Estivá, Itajubá, Minas Gerais 37500-279, Brazil

## Abstract

Considered a primary energy source, crude oil is essential for the production of industrial inputs. With the increase in investments in deepwater production systems, technological challenges emerge, especially in the exploitation of the Brazilian presalt, where the formation of hydrates and wax deposits can pose flow assurance risks. In addition to conditions that favor deposit formation, such as low seabed temperature, these fields also present a high gas/oil ratio and elevated pressures—factors that can alter the kinetics and thermodynamics of the phenomenon. This study aims to experimentally characterize the influence of three different gases (CO_2_, Natural Gas, N_2_, and N_2_ + *n*-hexane) at different pressures—up to 20.0 MPa gauge (MPag)— on the first and second crystallization events of a dead crude oil sample from the Brazilian presalt. The experiments were conducted using HPμDSC high-pressure cells, with pressure increased by the slow and gradual injection of each gas under study. It was found that N_2_ injection increases the WAT, while Natural Gas injection, in contrast, reduces the WAT. In the case of CO_2_ injection, pressure intervals were observed in which WAT increases, and others in which it remains constant; this behavior was also observed for the crystallization temperature of the second event.

## Graphical abstract

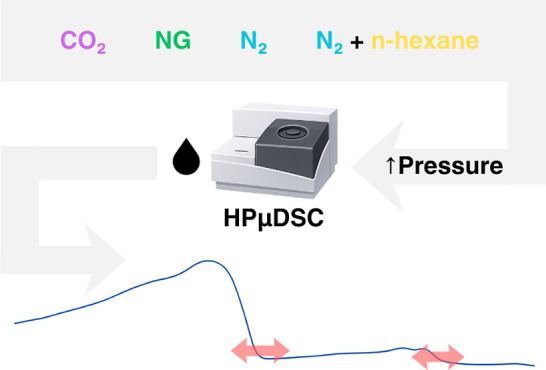

## Introduction

The production of crude oil and gas in deep waters presents major challenges related to flow assurance issues, especially when associated with wax deposition. These deposits, which gradually accumulate on the inner walls of the pipelines over production time, reduce the effective diameter through which the flow passes, increasing the pressure drop in the line and, consequently, increasing the energy consumption for pumping the extracted fluids and reducing well productivity.^1–4^ Depending on the severity of the deposition, the pipeline can be completely blocked and, in severe cases, result in accidents and environmental disasters. A thorough understanding of this problem is essential for its effective mitigation.^1,5,6^

Wax deposition occurs when two main factors are combined: crude oil composition and the temperature gradient. The extracted crude oil contains waxes in proportions greater than 4% of its molecular composition, and the production flow passes through a temperature gradient sufficient to reach the Wax Appearance Temperature (WAT), which is the temperature at which the waxes dissolved in crude oil begin to crystallize.^6–8^ Under this condition, small wax nuclei and aggregates are formed, which accumulate on the walls of the pipelines through a diffusion mechanism. Over time, these deposits grow and undergo a mechanism called aging, which increases the hardness of the deeper layers, making their removal difficult even with mechanical cleaning devices (pigs).^5,9^

There are several factors that influence the WAT of crude oil, as well as the characteristics of wax deposits. Pressure, temperature variation, flow rate, presence of water in the crude oil, and the presence and composition of gases are examples of factors that alter the wax deposition behavior.^5,7,8,10–12^ Although studies on wax deposition in crude oil have existed since the 1980s, the complexity and breadth of the topic, as well as the peculiarities of each reservoir, extraction conditions, and crude oil composition, more specific studies are still required for each application.^13^

In the deepwater crude oil production scenario, there are fields susceptible to flow assurance risks, especially when combined with high-pressure conditions, high CO_2_ concentrations, and low temperatures.^14,15^ In the Brazilian presalt scenario, there is also the challenge of a high gas-to-oil ratio (GOR), where the associated gas composition can reach CO_2_ contents of up to 80% mol.^15–17^

The high GOR and elevated CO_2_ content in the produced fluid typically lead to an increase in the size of the natural gas processing plant on the topside of the FPSO (Floating Production Storage and Offloading), often occupying more than half of the available space on the FPSO processing decks. Equally important is the need for reinjection of the CO_2_ removed from the produced natural gas, since environmental regulations do not permit its release into the atmosphere.^3,16^

Avoiding bringing part of the natural gas with high CO_2_ content to the surface processing plant not only reduces the area required for natural gas processing but also reduces the energy consumption associated with reinjection of CO_2_ into the producing reservoir. Studies have been conducted focusing on the separation and subsea reinjection of natural gas with high CO_2_ content.^16,18^

However, it should be noted that due to the high pressure in the subsea presalt scenario, the gas phase is in the form of dense gas, which reduces the density gradient compared to the other phases (crude oil and brine). This reduction in the density gradient decreases the gas–liquid separation velocity.

In gravity separators, it is expected that a small fraction of liquid—usually below 0.5% by volume—will be entrained in the separated gas stream. Likewise, a small fraction of gas is also expected to be entrained in the liquid stream (crude oil–brine mixture). The presence of liquids (crude oil–brine mixture) in the gas stream, even at low concentrations, can generate flow assurance issues in the gas stream to be reinjected, due to the risk of hydrate formation from the water presence, and wax deposition from the crude oil presence.

Hydrate formation is a thermodynamic phenomenon that can be mitigated by operating outside the generation region, or through continuous addition of thermodynamic or kinetic inhibitors. Hydrate formation also requires the presence of a minimum amount of free water in the gas stream. Generally, low levels of free water do not pose a risk of hydrate formation, except when accumulation points exist in the gas reinjection system. While wax deposition is also thermodynamically driven, it further depends on molecular diffusion mechanisms.

The wax deposition process is cumulative and occurs when, during production flow, the crude oil temperature is below the WAT. Therefore, crude oil flowing at temperatures above the WAT is not subject to wax deposition. On the other hand, crude oil flowing below the WAT presents a real risk of line and production facility obstruction due to the gradual accumulation of waxes.

In the case of deepwater flow of gas streams containing a small fraction of crude oil at temperatures below the WAT, there is also a risk of wax deposition, since the crude oil tends to adhere to the cold pipeline wall, initiating the wax formation process.

To determine the WAT, some well-established techniques exist in the literature, such as microcalorimetry (μDSC). Through thermogram analysis by cooling under isobaric conditions of crude oil samples, it is possible to identify the energy variation from the phase change of waxes and determine the onset crystallization temperature.^9^ This method offers high precision, for both dead and live crude oil samples, and is widely accepted in this type of analysis.^19^

High-pressure microcalorimetry (HPμ DSC) extends these capabilities to high-pressure conditions and is widely employed in phase-equilibria studies of gas–liquid and gas–solid systems, including hydrates, polymers, and complex petroleum fluids. HPμ DSC has demonstrated reliable determination of equilibrium temperatures and thermodynamic transitions under pressures up to 30 MPa, as shown in polymer–CO_2_ systems where phase-transition signatures and plasticization effects were accurately resolved despite strong gas-induced perturbations.^20^ Recent hydrate studies have further confirmed the method’s capability to detect subtle thermal events and provide high-fidelity thermodynamic data in gas-rich systems with pressure-sensitive equilibria, such as H_2_–DIOX and CH_4_-based hydrates, especially when combined with complementary spectroscopic characterization.^21^ In petroleum applications, HPμ DSC has been successfully applied to determine WAT and crystallization enthalpy under high pressure, although the reliability of the measurements depends strongly on experimental conditions such as gas saturation time, reference-cell pressurization, and baseline stability.^7^ Even with these limitations, the technique remains highly advantageous due to its sensitivity, short analysis time, and ability to resolve overlapping crystallization events, supporting its use for wax precipitation studies under deepwater and presalt pressure conditions.

It is worth noting that, by definition, the term WAT represents the temperature of the first wax crystallization event in each crude oil. However, other crystallization events may occur in crude oil. In the case of Brazilian postsalt and presalt reservoirs, many crude oil samples have been identified with two crystallization events. In this scenario, the second crystallization event—at a lower temperature—involves a much greater amount of waxes precipitating compared to the first event.

Many operators combine laboratory μDSC test results with field deposition histories to establish semiempirical correlations used to define the flow temperature adopted as the design basis of a given subsea crude oil and gas production system. In this case, it is not uncommon for the temperature of the second crystallization event, with the addition of a few degrees (e.g., 5 K), to be considered the “adjusted WAT” in design bases. For the sake of simplicity, the WAT (Wax Appearance Temperature) is assumed to correspond to the temperature at which the first crystallization occurs.

The determination of the WAT of Brazilian waxy crude oil by high-pressure micro calorimetry (HPμDSC) has shown that pressurization with light hydrocarbon-rich gas reduces the WAT, while pressurization with nitrogen increases it.^7,8^

However, Juyal et al.,^19^ focusing on the comparison of μDSC and HPμDSC results, did not observe significant changes in the WAT in experiments with model waxy oil in the presence of nitrogen under atmospheric conditions and 6.0 MPa, but did observe WAT reduction with increasing pressure in the presence of light hydrocarbons. This influence of light hydrocarbons was also observed using a coldfinger apparatus operating up to 6.0 MPa, where the deposited wax mass decreased with increasing pressure.^22^

Using a high-pressure rheometer, Helsper and Liberatore^23^ analyzed the influence of methane under pressures up to 10.0 MPa, along with wax concentration in crude oil, observing that WAT decreases with increasing pressure and increases with wax concentration.

Numerical analyses fitted with experimental parameters indicated that pressurization with methane and CO_2_ reduces the WAT of waxy crude oil, with the effect being more pronounced for CO_2_.^24^

Bidart et al.,^14^ also through thermodynamic modeling under high-pressure conditions and CO_2_ concentrations, observed WAT variations that increase or decrease depending on the pressure range.

Experimental μDSC analyses using Malaysian waxy crude oil in the presence of CO_2_ and pressures up to 5.0 MPa showed that increasing pressure reduces the WAT of the evaluated oil.^3^

Thus, the present study aimed to experimentally determine the influence of CO_2_ and natural gas under high pressures, up to 20.0 MPa, on the WAT of a Brazilian presalt crude oil. This work advances beyond previous literature by employing high-sensitivity HPμ DSC to provide unprecedented resolution of multiple crystallization events in Brazilian Presalt oils and new insights into wax behavior under supercritical CO_2_ conditions, complementing recent spectroscopic approaches.^25^ These results are extremely important for the development of subsea processing technologies, as they reveal how the wax deposition process behaves under high-pressure conditions and in the presence of CO_2_.

## Methodology

### Materials and Equipment

In this study, a stabilized crude oil (dead) sample representative of a Brazilian presalt field was used, provided by Petrobras. Upon receipt, the sample was heated to 353.15 K and homogenized in a sealed container to prevent the loss of light components. This procedure ensured not only the representativeness of the sample but also the elimination of its wax crystallization history. After this step, a smaller volume of approximately 300.0 mL was separated and stored in another sealed container for use in physicochemical characterization, SARA fractionation, chromatography, and microcalorimetry tests.

### Physicochemical Characterization

The following physicochemical parameters of the received dead crude oil were evaluated: absolute density and API gravity (ASTM D4052), kinematic and dynamic viscosity (ASTM D7042), and water content (ASTM E203-24). The results are shown in Table 1.

**Table 1:** Physicochemical Properties of the Analysed Dead Crude Oil

test	result	unit
API gravity	27.56	
absolute density (20 °C)	0.8850	g/cm^3^
kinematic viscosity (20 °C)	56.45	mm^2^/s
dynamic viscosity (20 °C)	49.96	mPa·s
water content	0.63	% (m/m)

### SARA

To obtain the SARA classification of the dead crude oil, an adapted version of the fractionation method based on ASTM D6560 and ASTM D3279 was employed. After the removal of asphaltenes according to ASTM D2007, the separation was carried out using open column chromatography (OPC). The saturated fraction was eluted with heptane, the aromatic fraction with a 7:3 (v/v) mixture of hexane and dichloromethane, and the resin fraction with a 1:1 (v/v) mixture of toluene and methanol. Based on the results presented in Table 2, the analyzed dead crude oil can be classified as mixed.

**Table 2:** SARA Fractionation Results^a^

test	volatiles (wt %)	asphalt. (wt %)	saturates (wt %)	aromatics (wt %)	resins (wt %)	retained polar^a^ (wt %)	total (wt %)
1	4.21	0.79	35.24	39.45	17.24	3.07	100.00
2	5.03	0.58	34.17	43.01	14.65	2.54	100.00
3	3.46	0.90	37.45	40.58	15.83	1.78	100.00
Average	4.23	0.76	35.62	41.01	15.91	2.46	100.00

a The percentage of retained polar compounds was not experimentally determined. It was theoretically calculated by subtracting the sum of the recovered fractions from the total mass (100%).

### Chromatographic Characterization

The dead crude oil was subjected to a separation process to isolate the saturates (SM), aromatics (AM), and polar (PM) fractions. The SAP fractionation (Saturates, Aromatics, and Polars) was performed using the OPC method. The saturate fraction was eluted with hexane, the aromatic fraction was eluted with a mixture of hexane and dichloromethane in a 7:3 (v/v) ratio, and the polar fraction was eluted with methanol.

The analysis was carried out by gas chromatography with a flame ionization detector (GC-FID), using an Agilent Technologies GC 7890 chromatograph equipped with a capillary column and an automatic injector.

The saturate fraction was injected into the chromatograph at a concentration of 20 μg/mL. A standard of *n*-alkanes ranging from C_7_ to C_40_, doped with *n*-C_20_H_42_ (*n*-eicosane), was analyzed for comparison of retention times.

The fractionation and GC-FID analysis processes were conducted in duplicate with appreciable repeatability. The chromatogram obtained for the saturate fraction (SM1) of the dead crude oil is shown in Figure 1.

**Figure 1:**
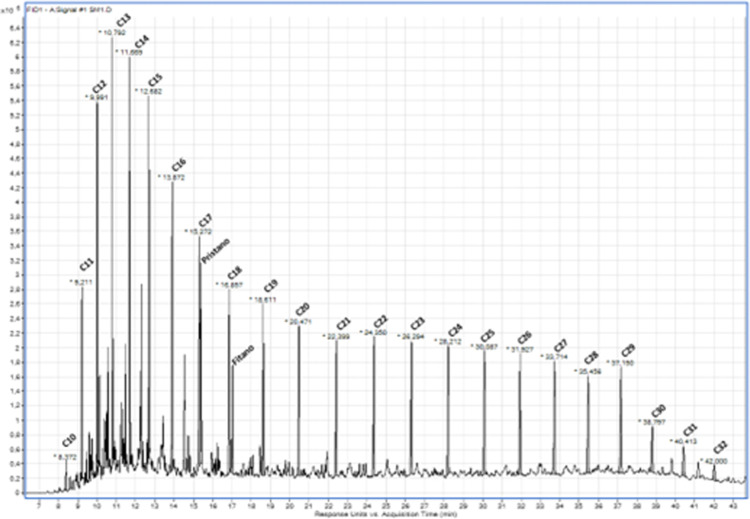
Chromatogram of the saturate fraction (SM1) from the Brazilian presalt dead crude oil sample used.

The quantification of the compounds was calculated based on the equation obtained from an analytical curve of *n*-eicosane with concentration points ranging from 5 to 500 μg/mL. The results are presented in Table 3.

**Table 3:** Determination of the Concentration of Compounds Identified by GC-FID in the Brazilian Pre-Salt Dead Crude Oil Sample

chain	concentration (mg/mL)
C_10_	0.030
C_11_	0.073
C_12_	0.114
C_13_	0.151
C_14_	0.155
C_15_	0.141
C_16_	0.134
C_17_	0.124
Pristane	0.111
C_18_	0.112
Phytane	0.084
C_19_	0.113
C_20_	0.105
C_21_	0.101
C_22_	0.106
C_23_	0.106
C_24_	0.107
C_25_	0.108
C_26_	0.106
C_27_	0.101
C_28_	0.094
C_29_	0.105
C_30_	0.059
C_31_	0.051
C_32_	0.040

### Microcalorimetry

Three different gases were used for pressurizing the test cells: natural gas (NG), carbon dioxide (CO_2_), and nitrogen (N_2_). NG and CO_2_ represent the main gases found in Brazilian presalt reservoirs, while N_2_, due to its low interaction with organic molecules, was employed to assess the isolated effect of pressure. The CO_2_ and N_2_ used had a purity grade of 99.0%. The composition of the NG used is shown in Table 4.

**Table 4:** Composition of the Natural Gas Mixture, Number of Moles, Partial Pressure, and Fraction Molar

component	number of moles	partial pressure (MPa)	concentration (mol %)
N_2_	3.0732	0.0259	0.5876
CO_2_	7.2185	0.0609	1.3802
CH_4_ (methane, C_1_)	468.0378	3.9492	89.4909
C_2_H_6_ (ethane, C_2_)	32.3319	0.2728	6.1820
C_3_H_8_ (propane, C_3_)	8.2074	0.0692	1.5693
*i*C_4_H_10_ (isobutane, *i*C_4_)	1.2045	0.0102	0.2303
*n*C_4_H_10_ (*n*-butane, *n*C_4_)	1.7787	0.0150	0.3401
*i*C_5_H_12_ (isopentane, *i*C_5_)	0.5246	0.0044	0.1003
*n*C_5_H_12_ (*n*-pentane, *n*C_5_)	0.3661	0.0031	0.0700
C_6_^+^	0.2568	0.0022	0.0491
total mixture	522.9994	4.4129	99.9998

High-pressure microcalorimetry (HPμDSC) tests were carried out using a MICROCALVET VII calorimeter from Setaram. The system operates with two 190.0 μL cells (one for the sample and one for the reference), both made of Hastelloy C276 and rated for pressures up to 100 MPa. The equipment was previously calibrated using naphthalene (C_10_H_8_) as a standard, following the manufacturer’s instructions.

To pressurize and maintain pressure during each test, a syringe pump from SYRUS and a pressure controller were used. This setup allows for a maximum pressure variation of 0.01 MPa in the sample cell by gradually displacing the syringe piston to control the gas volume introduced into the cell. Therefore, the experiments were conducted under quasi-isobaric conditions. The syringe pump was configured to be fed by the test gas cylinder and deliver pressure to both the sample and reference cells of the HPμDSC system. The configuration of the high-pressure microcalorimetry system is illustrated in Figure 2.

**Figure 2:**
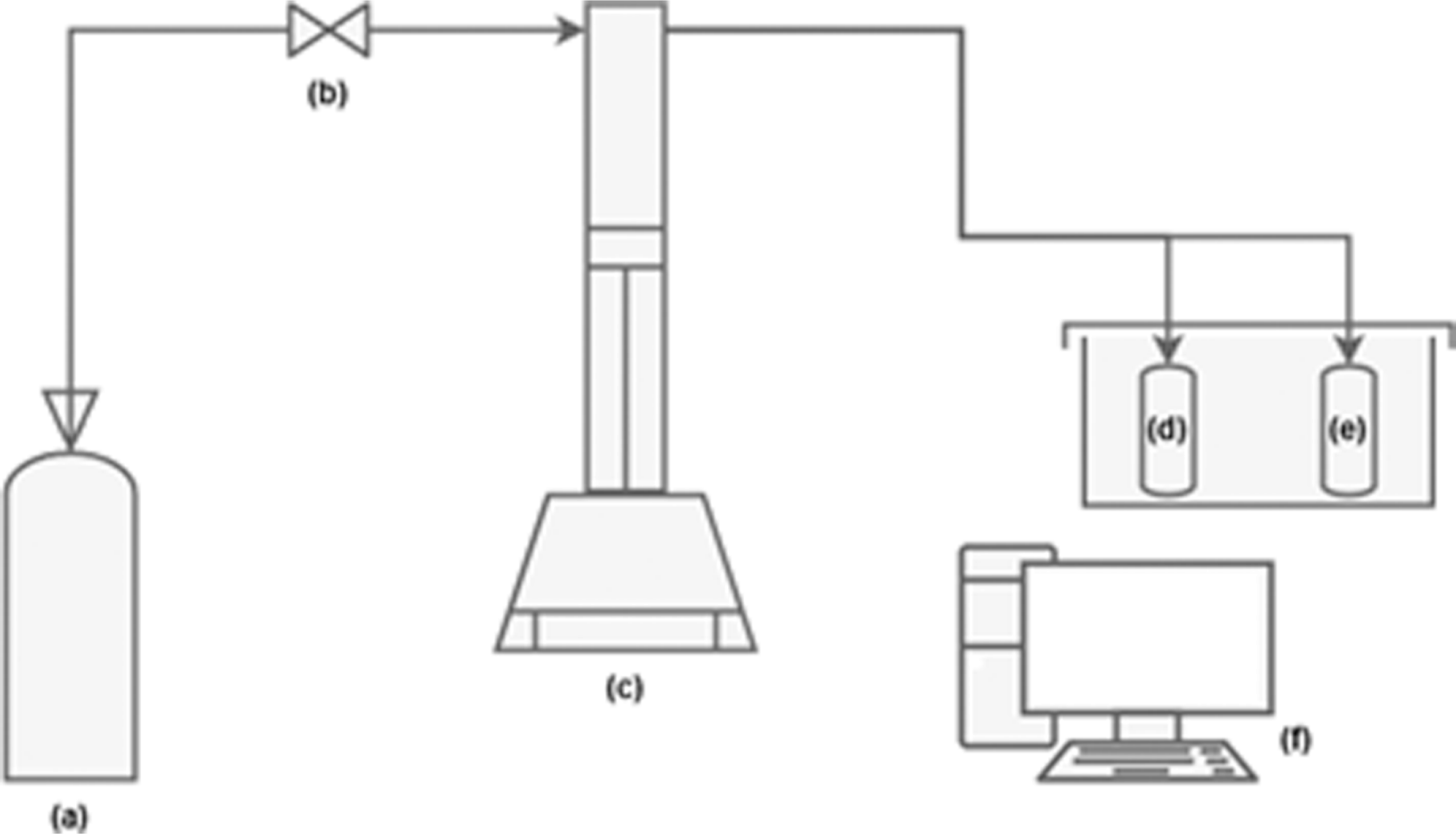
Configuration of the high-pressure microcalorimetry (HPμDSC) system. (a) Gas cylinder; (b) gas inlet valve; (c) syringe pump with pressure control; (d) sample cell coupled to the calorimeter; (e) reference cell coupled to the calorimeter; (f) data acquisition computer.

### Experimental Procedure

At the beginning of the microcalorimetry tests, the dead crude oil sample was heated and homogenized at 353.15 K for 4 h in a sealed container. This step ensured that all crystallized waxes were completely dissolved in the oil, providing a uniform sample for characterization.^7,8^ A small aliquot of the heated oil was then collected using a syringe and introduced into the HPμDSC test cell. The sample mass was quantified, the test cell was sealed, and then inserted into the calorimeter. A reference cell was also coupled to the device.

Before each calorimetric measurement at a given pressure, the thermal history of the oil in the test cell was reset by heating it to 353.15 K for 10 min. Subsequently, the temperature ramp was initiated. The ramp started at 353.15 K, and the temperature was decreased at a rate of 0.8 K/min until reaching 263.15 K. Finally, both the test and reference cells were reheated to 303.15 K.

This procedure was performed in the presence of different gases (N_2_, CO_2_, and NG) at pressures ranging from 0.1 to 20.0 MPag using the high-pressure microcalorimetry system (HPμDSC). All experiments were carried out in triplicate.

Additionally, experiments were conducted with the addition of 5 wt % of *n*-hexane (99.0% purity) to the dead crude oil to simulate the effect of the C_5_–C_10_ fraction, which represents the heavier hydrocarbons found in natural gas under subsea processing conditions on the WAT. These specific tests were performed using N_2_ as the pressurization medium.

The experimental procedure adopted is described as follows.

1. Heating and homogenization of the dead crude oil sample.
2. Collection of the dead crude oil sample using a syringe and subsequent weighing.
3. Addition of the sample to the HPμDSC test cell.
4. Pressurization to 0.1 MPag.
5. Heating of the sample in the HPμDSC cell to 353.15 K for 10 min to eliminate any crystallization history and ensure gas phase solubilization.
6. Execution of the test ramp at 0.1 MPag.
7. Pressure increase to the new test condition (2.5, ..., 20.0 MPag).
8. Heating of the sample in the HPμDSC cell to 353.15 K for 10 min to eliminate any crystallization history and ensure gas phase solubilization.
9. Execution of the test ramp at the new test condition.
10. Repeat steps 7, 8, and 9 until completing the intended test matrix.
11. Slow depressurization of the system.

Finally, a microcalorimetric analysis of the dead crude oil was also performed at atmospheric pressure in the presence of air to compare this result with those obtained under different pressures with N_2_, CO_2_, and natural gas.

### Data Processing

The results obtained from the microcalorimetry tests were processed using the Microvelt 2.1 software from Setaram. Data acquisition and baseline correction were performed following established methodologies.^26^

To ensure accuracy in determining the onset of the crystallization event, measures were taken to prevent the occurrence of systematic bias during the analysis: control of experimental conditions, equipment calibration, and blank measurement.

The microcalorimetry system features robust thermal insulation and a thermostatic bath that maintains uniform temperature across the equipment, while the calorimetric furnace varies the temperature within the specified range without thermal interference, ensuring strict control of experimental conditions.

The equipment was calibrated using a primary standard of naphthalene, provided by the manufacturer and sealed in a hermetic stainless steel cell. During the tests, four calibrations were performed using the naphthalene standard, which showed a maximum deviation of +0.063 K for temperature and +0.394 kJ/mol for fusion enthalpy.

The blank test was conducted at atmospheric pressure using empty reference and sample cells, applying the same temperature ramps and cooling rates as in the experiments. The subtraction of the blank signal from the experimental signal was performed automatically by the software. This subtraction eliminates background interference, resulting in a corrected thermogram that exclusively represents the sample response.

Figure 3 presents the thermograms of the blank test (a), the raw test (b), and the baseline (c). The selected condition was the nitrogen +5 wt % of *n*-hexane test at 2.5 MPag.

**Figure 3:**
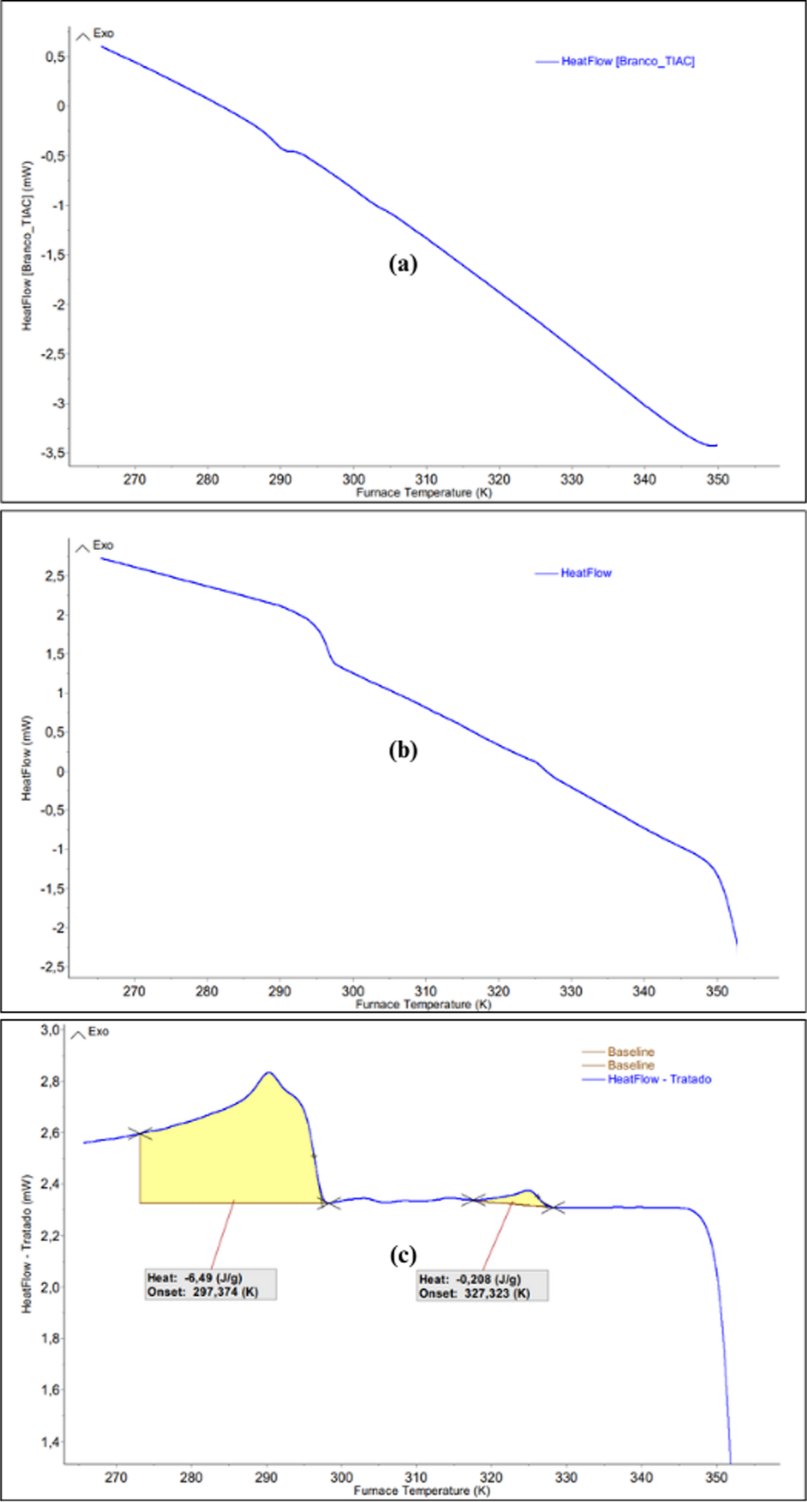
Data treatment procedures for the thermogram of dead crude oil with N_2_ + 5 wt % of *n*-hexane at 2.5 MPa. (a) Blank measurement, (b) raw crude oil thermogram, and (c) determination of crystallization temperatures.

The determination of the onset temperature of crystallization events is performed automatically by the software algorithm. After the left and right boundaries of the peak are manually marked, the onset and offset are defined at the inflection points according to the selected baseline type, and the tangents are automatically adjusted. A tangential baseline was adopted for the first event and a horizontal baseline for the second event, both originating from the first (left) point, to ensure precision in determining the onset of the phase changing regarding to the wax.

All data plots were generated using the VEUSZ software. Error bars represent the standard deviation of triplicate measurements. The differences in crystallization temperatures between conditions were calculated, and the associated uncertainties were propagated accordingly.

## Results and Discussion

### Thermogram of the Dead Crude Oil at Atmospheric Pressure

The heat flow versus temperature curve (thermogram) of the dead crude oil evaluated at atmospheric pressure and in the presence of air is shown in Figure 4. As can be observed, this dead crude oil exhibits two crystallization events. The first event, at 328.18 ± 1.49 K, represents a small energy variation when compared to the second crystallization event at 298.49 ± 1.05 K. Thus, it can be stated that the amount of paraffins precipitating during the second event is significantly higher than during the first crystallization event.

**Figure 4:**
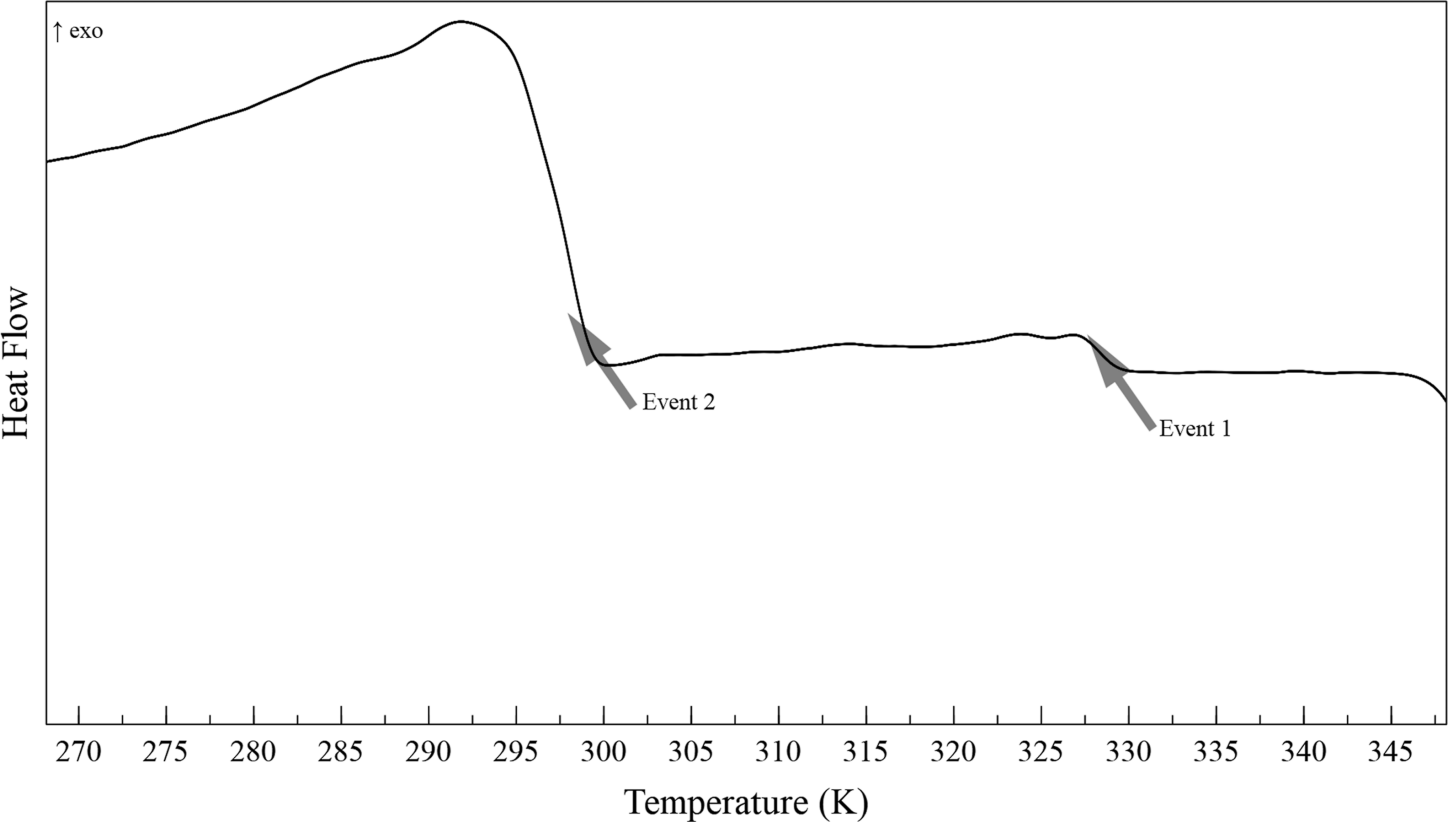
Dead crude oil thermogram at atmospheric pressure and in the presence of air, obtained in the high-pressure cell.

It is worth noting that the paraffins precipitating in the first event act as nucleation sites for the paraffins that precipitate during the second crystallization event, which in practice accelerates the crystal growth process associated with the second event.

The existence of two distinct crystallization events has also been documented in other Brazilian crude oils.^27,28^

### Influence of Pressure and Gas Phase Composition on the Temperature of the First and Second Crystallization Events

Table 5 reports the mean values and standard deviations of the temperatures obtained from three replicate experiments under each condition, showing that the repeatability in determining the onset of the wax crystallization event is satisfactory. The tests with carbon dioxide exhibited larger variations, which can be attributed to the high sensitivity of the fluid’s properties (e.g., density, solvation power) to minor fluctuations in pressure and temperature near its critical point, making the thermal signal and baseline less stable. The determination procedure followed the same methodology as for the other gases but was not carried out at pressures where the energy variation of the gas was greater than that associated with the phase transition of the wax crystals.

**Table 5:** Onset Temperatures (K) of the First and Second Crystallization Events under Different Gas Atmospheres and Pressures^a^

pressure (MPa)	gas	first event temperature (K)	second event temperature (K)
0.1	N_2_	328.26 ± 0.71	298.52 ± 0.24
0.1	N_2_ + *n*-hexane	327.75 ± 0.35	297.44 ± 0.14
0.1	natural gas	327.71 ± 0.96	298.29 ± 0.15
0.1	CO_2_	328.10 ± 1.06	298.26 ± 0.43
2.5	N_2_	328.53 ± 0.69	298.83 ± 0.18
2.5	N_2_ + *n*-hexane	327.54 ± 0.17	297.56 ± 0.14
2.5	natural gas	327.63 ± 0.46	298.12 ± 0.23
2.5	CO_2_	326.52 ± 0.74	296.85 ± 0.24
5.0	N_2_	328.85 ± 0.60	299.07 ± 0.18
5.0	N_2_ + *n*-hexane	327.76 ± 0.26	297.84 ± 0.14
5.0	natural gas	327.23 ± 0.35	297.71 ± 0.53
5.0	CO_2_	324.66 ± 0.64	294.91 ± 0.02
7.5	N_2_	329.08 ± 0.47	299.35 ± 0.18
7.5	N_2_ + *n*-hexane	328.09 ± 0.05	298.12 ± 0.12
7.5	natural gas	326.25 ± 0.74	296.88 ± 0.91
7.5	CO_2_	–	–
10.0	N_2_	329.41 ± 0.48	299.64 ± 0.17
10.0	N_2_ + *n*-hexane	328.33 ± 0.22	298.34 ± 0.14
10.0	natural gas	326.22 ± 0.45	296.78 ± 0.79
10.0	CO_2_	–	–
12.5	N_2_	329.73 ± 0.52	299.97 ± 0.16
12.5	N_2_ + *n*-hexane	328.56 ± 0.14	298.62 ± 0.18
12.5	natural gas	325.29 ± 0.63	296.02 ± 0.86
12.5	CO_2_	–	295.02 ± 0.26
15.0	N_2_	330.01 ± 0.56	300.24 ± 0.15
15.0	N_2_ + *n*-hexane	328.93 ± 0.21	298.95 ± 0.15
15.0	natural gas	324.85 ± 0.76	295.68 ± 0.96
15.0	CO_2_	321.71 ± 1.55	295.05 ± 0.55
20.0	N_2_	330.95 ± 0.47	300.98 ± 0.21
20.0	N_2_ + *n*-hexane	329.60 ± 0.29	299.62 ± 0.17
20.0	natural gas	324.28 ± 0.91	295.52 ± 0.94
20.0	CO_2_	322.55 ± 1.80	295.81 ± 0.85

a Values represent: mean ± standard deviation.

### Pressurization with Nitrogen (N_2_)

Figure 5 shows the temperatures of the first and second crystallization events as a function of crude oil pressurization with nitrogen, from 0.1 to 20.0 MPag. Under the evaluated thermodynamic conditions, nitrogen remains in the gaseous phase. From the analysis of this figure, it is observed that nitrogen pressurization gradually increases the temperature of both crystallization events: from 328.26 ± 0.71 to 330.95 ± 0.47 K (difference of +2.688 ± 0.849 K) for the first event and from 298.52 ± 0.24 to 300.98 ± 0.21 K (+2.46 ± 0.32 K) for the second event.

**Figure 5:**
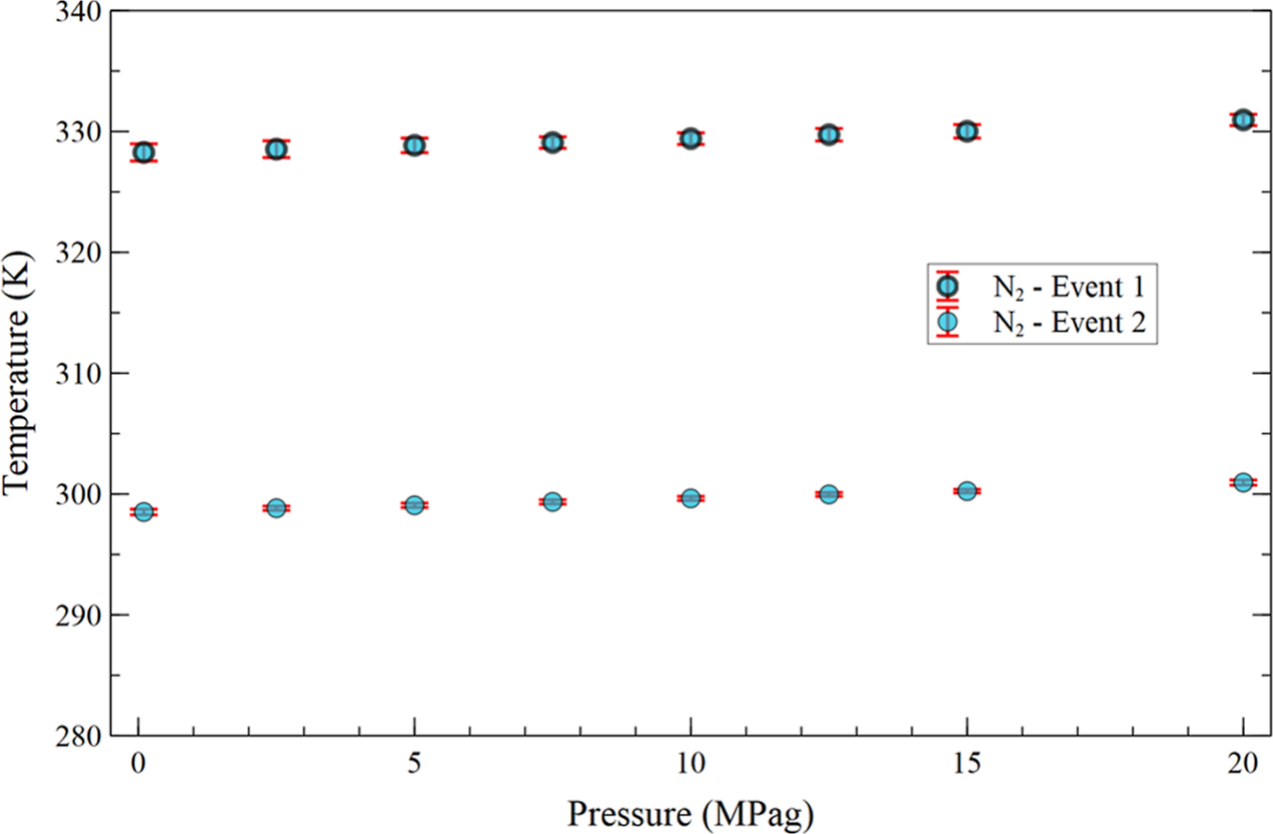
Average temperature of the first and second crystallization events as a function of nitrogen pressurization from 0.1 to 20.0 MPag.

This behavior can be explained by nitrogen’s higher affinity for the lighter nonpolar fractions of crude oil compared to the heavier nonpolar fractions, such as paraffins. For comparison purposes, studies conducted by Battino, Rettich, and Tominaga^29^ show that nitrogen’s solubility in *n*-hexane (*n*-C_6_) is about twice as high as in *n*-hexadecane (*n*-C_16_). As nitrogen dissolves into the crude oil, light-intermediate alkane molecules (C_2_ to C_10_), which are good paraffin solvents, become less available to interact with heavier paraffin molecules, leading to an increase in the precipitation temperature of these paraffins, as experimentally observed by the rise in the crystallization event temperatures. Therefore, the behavior of nitrogen under pressure serves as a base case for comparing the effects of other gases in the subsequent plots.

#### Pressurization with Nitrogen (N_2_) and Addition of *n*-Hexane (*n*-C_6_)

To evaluate the effect of intermediate-chain *n*-alkanes on the temperature of the first and second crystallization events, 5 wt % of *n*-hexane (*n*-C_6_) was premixed with the crude oil, and the nitrogen pressurization test was repeated from 0.1 to 20.0 MPag. Due to its affinity for nonpolar molecules, *n*-hexane is considered a good paraffin solvent. Under the tested thermodynamic conditions, nitrogen remained in the gaseous phase.

The results are shown in Figure 6. From the figure, it can be seen that nitrogen pressurization from 0.1 to 20.0 MPag, in the presence of 5 wt % *n*-hexane, raises the crystallization temperatures from 327.75 ± 0.35 to 329.60 ± 0.29 K (+1.85 ± 0.46 K) for the first event, and from 297.44 ± 0.14 to 299.62 ± 0.17 K (+2.18 ± 0.22 K) for the second event.

**Figure 6:**
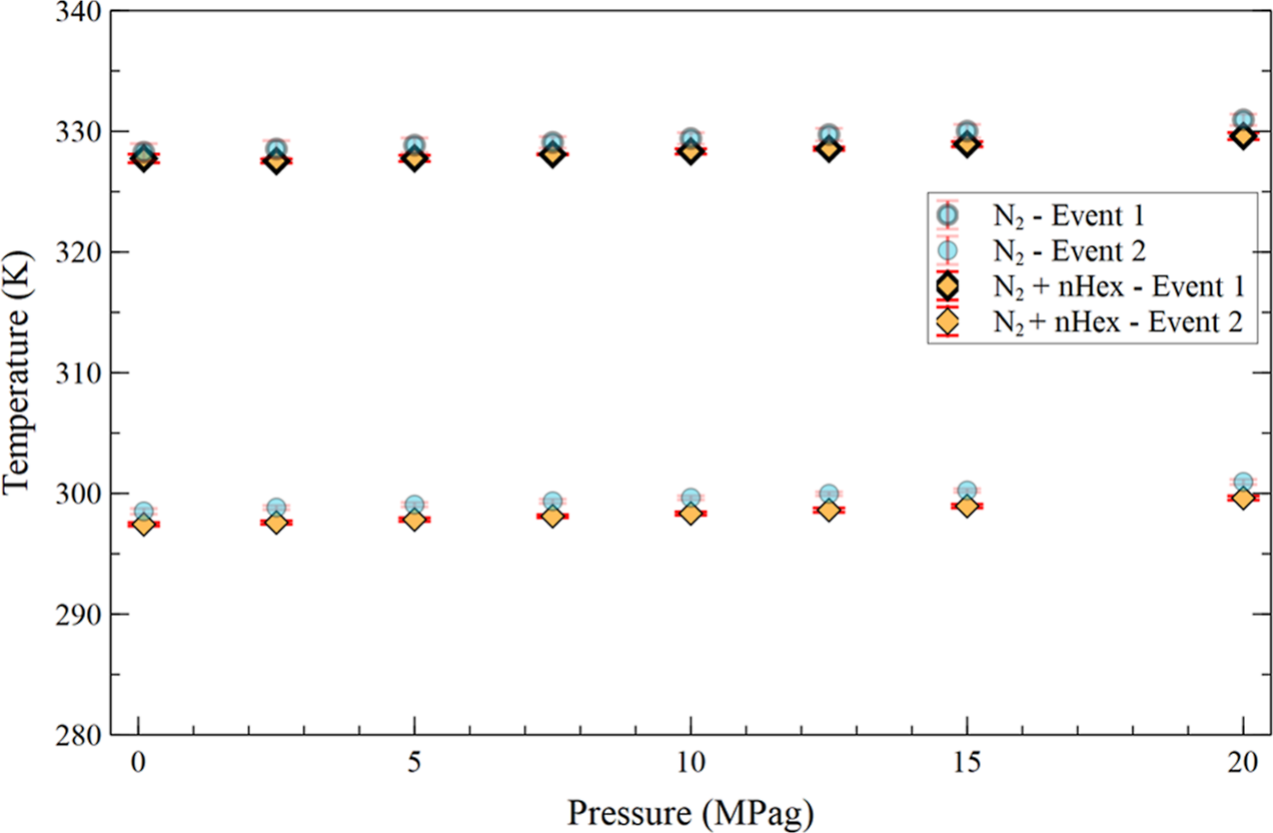
Average temperature of the first and second crystallization events as a function of nitrogen pressurization from 0.1 to 20.0 MPag with the addition of 5 wt % *n*-hexane to the crude oil and N_2_ (base case).

It can thus be concluded that even with the addition of 5 wt % of *n*-hexane, it is not possible to completely cancel out the effect of nitrogen pressurization on the increase in crystallization temperatures. However, the observed temperatures remained slightly lower than those seen when pressurization was conducted with nitrogen alone, within the range of 0.1 to 20.0 MPag.

#### Pressurization with Natural Gas (NG)

Figure 7 shows the temperatures of the first and second crystallization events as a function of crude oil pressurization with commercial natural gas from 0.1 to 20.0 MPag. For comparison, the results for nitrogen are also plotted. Under the evaluated conditions, the commercial natural gas remains in the gaseous phase.

**Figure 7:**
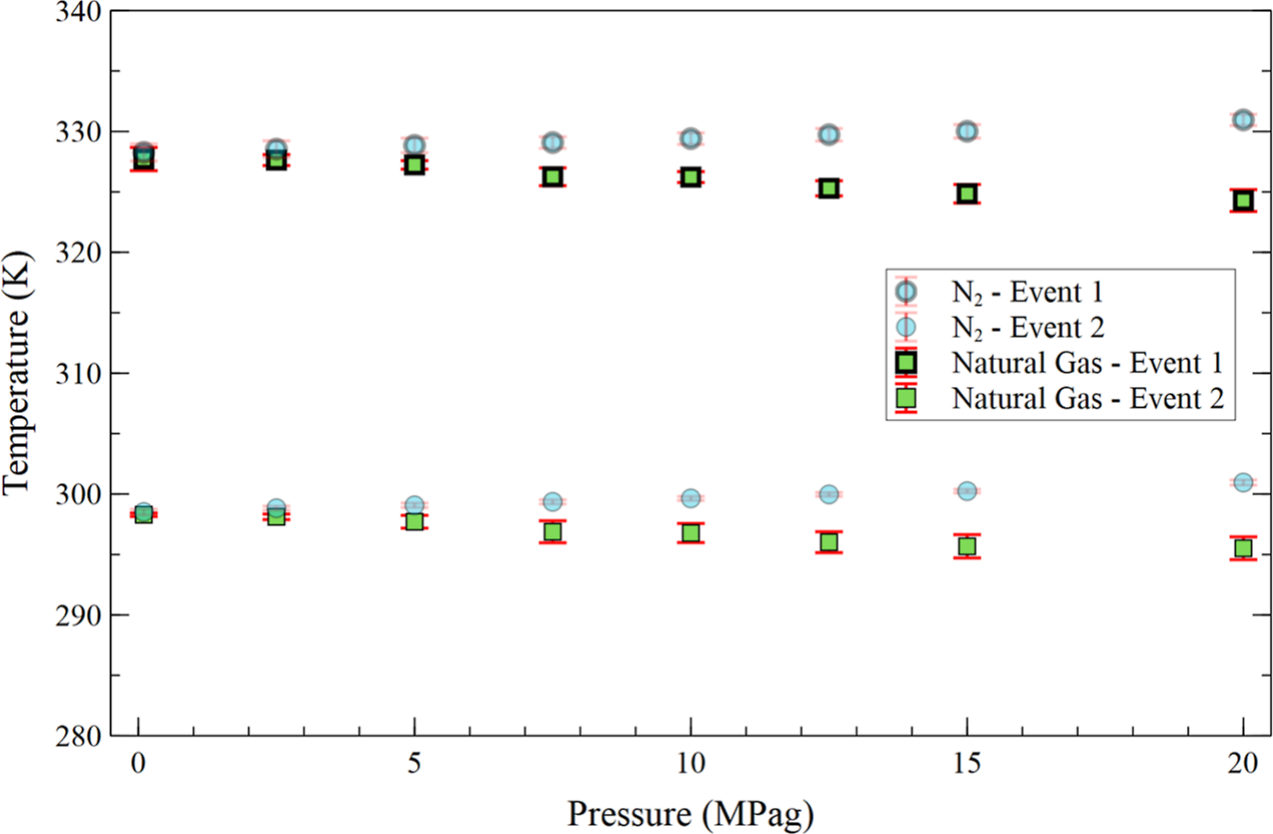
Average temperature of the first and second crystallization events as a function of commercial natural gas pressurization and N_2_ (base case) from 0.1 to 20.0 MPag.

From the analysis of the figure, it is observed that pressurization with natural gas gradually lowers the temperature of both crystallization events: from 327.71 ± 0.69 to 324.28 ± 0.906 K (−3.43 ± 1.14 K) for the first event, and from 298.29 ± 0.15 to 295.52 ± 0.94 K (−2.77 ± 0.96 K) for the second event.

This behavior is associated with the affinity of nonpolar components of natural gas (mainly CH_4_, C_2_H_6_, and C_3_H_8_) for the nonpolar paraffin molecules. As pressure increases, more of these components dissolve into the crude oil, leading to lower temperatures for the crystallization events. It is worth noting that the commercial natural gas used contained only a small amount of heavier alkanes (C_4+_ < 0.8% mol), which have greater affinity and solvency for paraffins.

### Pressurization with Carbon Dioxide (CO_2_)

The dead crude oil pressurization tests with carbon dioxide were conducted in two pressure ranges: from 0.1 to 5.0 MPag and from 12.0 to 20.0 MPag.

The exclusion of the 5.0 to 10.0 MPag range was due to the proximity to the critical point of CO_2_ (7.39 MPa and 304.25 K), where the fluid’s density fluctuations and transitional behavior prevented a reliable and clear interpretation of the μDSC signal.

At pressures near the critical point of CO_2_ (7.5 MPag), the thermal signal from the fluid’s phase transitions overshadowed the wax crystallization events, making it not possible to reliably determine the crystallization temperatures.

Figure 8 presents the temperatures of the first and second crystallization events as a function of crude oil pressurization with CO_2_ from 0.1 to 5.0 MPag. Analysis shows that CO_2_ pressurization lowers the crystallization temperatures from 327.65 ± 1.06 to 324.66 ± 0.64 K (−3.44 ± 1.24 K) for the first event, and from 298.68 ± 0.43 to 294.91 ± 0.02 K (−3.493 ± 0.433 K) for the second event. In this pressure range, while bulk CO_2_ remains in the gaseous state, it exhibits significant and increasing solubility in crude oil with rising pressure, as demonstrated by Mosavat et al.^30^ The dissolution of CO_2_ into the oil phase may be the primary mechanism responsible for the reduction in crystallization temperatures observed in this range.^31^

**Figure 8:**
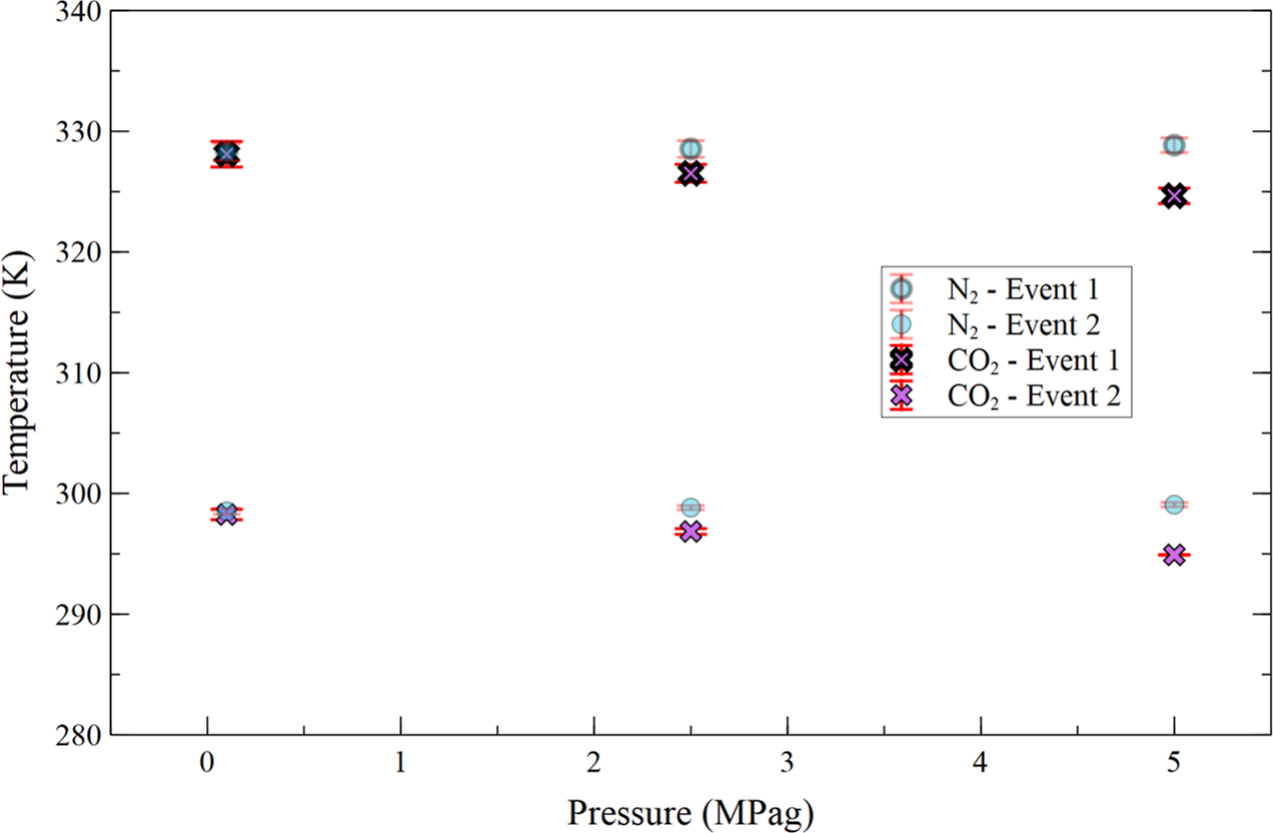
Average temperature of the first and second crystallization events as a function of CO_2_ and N_2_ (base case) pressurization from 0.1 to 5.0 MPag.

Figure 9 shows the temperatures of the first and second crystallization events as a function of crude oil pressurization with CO_2_ from 10.0 to 20.0 MPag. In this pressure range and for temperatures above 304 K, CO_2_ is a supercritical fluid that behaves like a dense gas, and the measured crystallization temperatures exhibited no statistically significant trend. The calculated crystallization temperatures were +0.84 ± 2.38 K for the first event and +0.79 ± 0.89 K for the second event, indicating that the observed changes lie within the experimental uncertainty. Therefore, crystallization temperatures can be considered constant within this pressure range.

**Figure 9:**
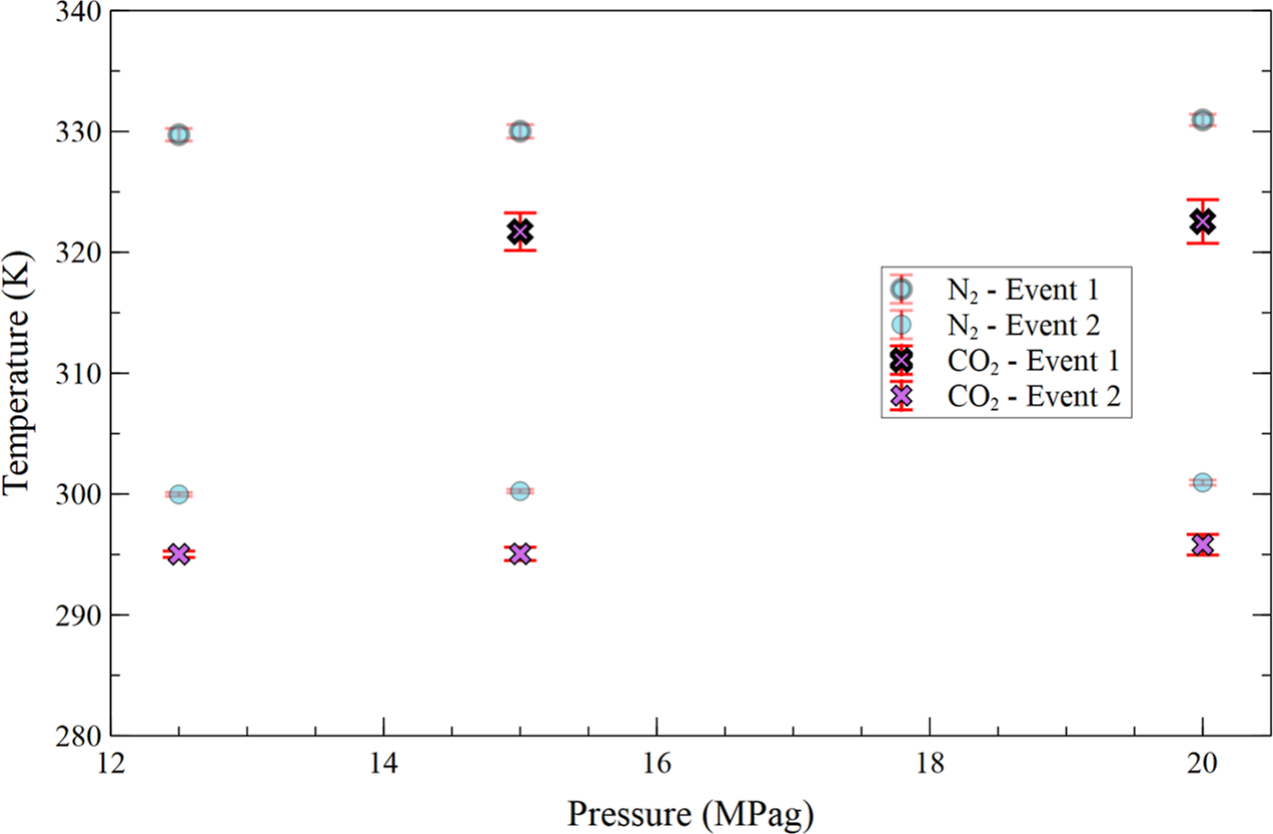
Average temperature of the first and second crystallization events as a function of CO_2_ and N_2_ (base case) pressurization from 12.0 to 20.0 MPag.

Mosavat et al.^30^ also demonstrated that the oil swelling factor (SF) increases up to the pressure corresponding to the CO_2_ phase transition. At equilibrium pressures beyond this point, the CO_2_–oil interaction is primarily governed by the extraction of the remaining light to medium hydrocarbons rather than by oil swelling. Consequently, the oil phase begins to contract, leading to a reduction in the swelling factor. This behavior was attributed to the formation of a high-density CO_2_-rich phase with an enhanced ability to extract light to medium hydrocarbon components from the crude oil, thereby offsetting part of the CO_2_ solvency effect on the crystallization temperatures.

The most significant finding of this study is the net effect of CO_2_ across the entire pressure range (0.1 to 20.0 MPag). A substantial and statistically significant reduction in crystallization temperatures is observed: from 328.10 ± 1.06 to 322.55 ± 1.80 K (−5.55 ± 2.09 K) for the first event, and from 298.41 ± 0.43 to 295.81 ± 0.85 K (−2.60 ± 0.96 K) for the second event. This overall decrease confirms that the solvent effect of CO_2_ is the dominant phenomenon governing the system’s behavior.

However, the trajectory of this reduction exhibits a nonlinear behavior. The nearly constant crystallization temperatures observed within the supercritical pressure range (10.0–20.0 MPag) suggest the predominance of a competing mechanism, namely the extraction of light to medium hydrocarbon components, which mitigates the solvent effect of CO_2_.

We also propose that light to intermediate hydrocarbons (C_5_–C_10_) act as natural solvents (peptizers) for heavier, amphiphilic molecules such as resins and asphaltenes. Their selective extraction by CO_2_ disrupts the colloidal equilibrium of the crude oil, potentially leading to asphaltene flocculation and the formation of colloidal aggregates. These aggregates may subsequently serve as effective nucleation sites for paraffin crystallization, thereby interfering with the crystallization process and counteracting the solvent effect of CO_2_ on paraffin molecules.

This mechanism is strongly supported by the findings of Shi et al.,^32^ who demonstrated that multiple cycles of CO_2_ injection preferentially extract light and intermediate components (C_2_–C_10_), thereby enriching the residual oil in heavier fractions (C_11+_) and promoting asphaltene precipitation and flocculation. Consequently, the competition between CO_2_ solvation (which lowers the crystallization temperature) and this nucleation mechanism (which increases it) contributes to the stabilization of crystallization temperatures in the supercritical regime. Nevertheless, the pronounced net reduction observed from 0.1 to 20.0 MPag unequivocally indicates that the solvation effect of CO_2_ predominates over the entire pressure range.

### Discussion on the Use of Dead Oil and Implications for Live Oil Systems

It is well-established in the literature that the use of dead oil, as in this study, presents limitations when directly extrapolating results to live oil in flowlines or reservoirs.^7,8,19^ Live oil, saturated with reservoir gases under high pressure, typically exhibits a lower crystallization temperature due to the solubilizing effect of light ends (C_1_–C_7_) on wax.

In this experimental setup, gas dissolution into the dead crude oil inside the sealed HPμ DSC cell occurs under controlled pressure, creating a gas-saturated oil system that allows investigation of gas effects on wax crystallization. However, the initial absence of these light ends in the dead oil means the mixture’s composition differs from authentic live oil, potentially leading to a higher measured WAT compared to the true live oil value.

If these tests were to be repeated with authentic live oil, we would anticipate a further reduction in the WAT for all gases tested, particularly for natural gas and CO_2_, as the light-intermediate hydrocarbons and CO_2_ would act as additional solvents. The observed trends—N_2_ increasing WAT and hydrocarbon gases/CO_2_ decreasing it—would likely remain valid, but the absolute temperature values would be shifted downward. Furthermore, the potential for partial loss of volatile components during sample handling, even from sealed containers, is a known challenge and could contribute to a slight overestimation of the WAT. Therefore, the data presented here should be interpreted as a conservative estimate (higher WAT) for flow assurance design purposes, highlighting the critical effect of gas composition under high pressure.

## Conclusions

This study demonstrated that gas composition and pressure critically influence the wax crystallization temperatures of a Brazilian presalt dead crude oil. Nitrogen pressurization increased the temperatures, while natural gas and subcritical CO_2_ reduced them. However, a key finding was the stabilization of the crystallization temperature under supercritical CO_2_ conditions (10.0–20.0 MPag), suggesting the emergence of a competing mechanism where the extraction of light-intermediate hydrocarbons counteracts the solvent effect of CO_2_.

Beyond these specific findings, our work provides several distinct advancements to the field: (i) Novel Detection of Multiple Crystallization Events: Using high-sensitivity HP μ DSC, we identified two distinct crystallization temperatures in Brazilian Presalt crude oils, revealing a more complex crystallization behavior than previously reported with single-point WAT detection methods like FT-IR.^25^ (ii) Comprehensive Study on Brazilian Presalt Oils: Our work provides the detailed characterization of wax crystallization under high-pressure gases for this economically critical petroleum system. (iii) New Insight into Supercritical CO_2_ Behavior: We discovered and mechanistically explained the crystallization temperature stabilization under supercritical CO_2_ conditions (10.0–20.0 MPag), revealing the competition between CO_2_ solvation and light hydrocarbon extraction. (iv) Direct Thermodynamic Measurements: unlike spectroscopic methods, DSC directly measures the heat flow associated with phase transitions, providing fundamental thermodynamic data on the crystallization process. While previous studies have confirmed the WAT-suppressing effects of CO_2_ and natural gas, our work provides a more fundamental understanding through higher-resolution detection of complex crystallization behavior and new mechanistic insights specific to Presalt petroleum systems.

While the trends observed are expected to hold for live oil, future work should focus on direct characterization of asphaltene behavior under high pressure to further elucidate the precise mechanisms identified in this study. These findings underscore the critical importance of considering both gas composition and pressure in flow assurance strategies.

## Supplementary Material



## Notes

Table of each Temperature Crystallization. The complete data set of all crystallization temperature measurements from triplicate experiments is available as an Excel spreadsheet in the Supporting Information files. File: Complete_Crystallization_Data set.xlsx The spreadsheet contains the following sheets:Sheet 1: Baseline measurements (oil without gas). Sheet 2: CO_2_ pressurization data. Sheet 3: N_2_ pressurization data. Sheet 4: Natural Gas pressurization data. Sheet 5: N_2_ + *n*-Hexane mixture data. All temperatures are reported in Celsius values (XLSX)
